# Science and technical priorities for private sector action to address biodiversity loss

**DOI:** 10.1098/rstb.2023.0208

**Published:** 2025-01-09

**Authors:** Emily J. McKenzie, Matt Jones, Nina Seega, Juha Siikamäki, Varsha Vijay

**Affiliations:** ^1^ Taskforce on Nature-related Financial Disclosures (TNFD), 24 Holborn Viaduct, London EC1A 2BN, UK; ^2^ UN Environment Programme World Conservation Monitoring Centre (UNEP-WCMC), Cambridge CB3 0DL, UK; ^3^ University of Cambridge Institute for Sustainability Leadership (CISL), Cambridge CB2 1GG, UK; ^4^ International Union for Conservation of Nature (IUCN), Washington, DC 20090, USA; ^5^ Science Based Targets Network (SBTN), New York, NY 10008-7082, USA

**Keywords:** business, finance, natural capital accounts, nature data, nature measurement, risk assessment

## Abstract

Target 15 of the Kunming–Montreal Global Biodiversity Framework recognizes the importance of the private sector monitoring, assessing and disclosing biodiversity-related risks, dependencies and impacts. Many businesses and financial institutions are progressing with science-based assessments, targets and disclosures and integrating into strategy, risk management and capital allocation decisions. Developments will continue in response to investor expectations, emerging corporate sustainability reporting regulations in Europe, China and elsewhere and evolving global sustainability reporting standards. Voluntary action is also being encouraged by the disclosure recommendations of the Taskforce on Nature-related Financial Disclosures and the target-setting methods of the Science Based Targets Network. Based on experience supporting the private sector in practice, we identify four critical science and technical advances needed to enable business action at scale and to redirect finance globally to halt and reverse biodiversity loss. First, consensus on indicators and metrics for measuring changes in the state of nature and provision of ecosystem services. Second, access to global, regularly updated, location-specific and consistent nature data. Third, standardized and consistent accounting systems that structure data, support risk management and create accountability at corporate, ecosystem and national levels. Fourth, integrated risk assessment approaches to help corporates, financial institutions, central banks and supervisors to assess nature-related risks.

This article is part of the discussion meeting issue ‘Bending the curve towards nature recovery: building on Georgina Mace's legacy for a biodiverse future’.

## 1. Introduction

Biodiversity is declining faster than at any time in human history [1–3]. This rapid biodiversity loss is increasingly recognized as a significant global risk, including in the private sector. This is notable through the marked change in the regularly published World Economic Forum Global Risks Report. In 2006, biodiversity was considered an ‘outlier’ that ‘didn’t make it onto the watch list’ [4]. Since then, the perception of the importance of climate and environmental risks has increased dramatically. In 2024, five of the top ten risks identified over the following decade were environmental, including biodiversity loss and ecosystem collapse, natural resource shortages and pollution as well as risks related to climate change [5]. Concerningly, these risks are still perceived to be those for which society generally, and businesses specifically, are the least prepared [6].

Action by businesses and financial institutions, alongside governments, intergovernmental organizations, civil society and communities, will be vital to halting and reversing biodiversity loss. In recognition of this, the Kunming–Montreal Global Biodiversity Framework (GBF) promotes a ‘whole of society’ approach [7]. The GBF was adopted as part of a package of decisions that also introduced a new monitoring framework [8]. While the headline indicators are established, there are significant gaps in the lower tiers of indicators, including those relating to private sector action. The finance sector has a particularly important role in redirecting private financial flows away from activities that harm nature and towards conservation, restoration and sustainable use [9]. Achieving the goals and targets of the GBF will also require businesses to improve the sustainability of production activities along value chains.

Governments recognized in the GBF the importance of businesses identifying, assessing and reporting on biodiversity, ‘in order to progressively reduce negative impacts on biodiversity, increase positive impacts, reduce biodiversity-related risks to business and financial institutions, and promote actions to ensure sustainable patterns of production’ [7]. Target 15 states that parties to the Convention on Biological Diversity should take measures to encourage and enable large and transnational companies and financial institutions to ‘regularly monitor, assess and transparently disclose their risks, dependencies and impacts on biodiversity’ [7]. Target 15 can support target 19 ‘to substantially and progressively increase the level of financial resources for biodiversity from all sources, in an effective, timely and easily accessible manner’ [7]. This financial capital can enable the investment in biodiversity needed to achieve the agreement’s global goals and vision.

Global standards and regulations for corporate biodiversity disclosure are now emerging. In Europe, there are now mandatory corporate sustainability reporting standards that include biodiversity and ecosystems, following the European Corporate Sustainability Reporting Directive [10]. In February 2024, three major stock exchanges in China announced their draft mandatory disclosure requirements for consultation, which include biodiversity [11]. Further biodiversity standards and regulations are expected to emerge in other jurisdictions in response to both target 15 and the expected integration of biodiversity into the global sustainability reporting baseline. In June 2023, the International Sustainability Standards Board (ISSB) published inaugural global sustainability disclosure standards [12]. There was support in response to the ISSB’s public consultation on including a new project on ‘biodiversity, ecosystems and ecosystem services’ towards a thematic global standard [13]. The Global Reporting Initiative (GRI) has updated its biodiversity standard, to meet the information needs of stakeholders focused on impacts [14].

A number of initiatives have stimulated and supported voluntary action on biodiversity by corporates and financial institutions [15–19]. These build on decades of advances in interdisciplinary science and research on biodiversity, Earth systems, ecosystem services and conservation, including framework development, synthesis reviews and global, regional and thematic assessments. These have been translated to the language, processes, methods, decision support needs and priorities of the private sector. Research advances have been particularly important to establish key concepts [9,20], demonstrate change over time in biodiversity, nature, ecosystem services and Earth systems, and establish the connections among these [21–25], provide classification systems [26], set out approaches to measurement [27], establish natural capital accounting and assessment systems [16,28], provide methods for target-setting and measuring performance [18,29] and make the business case for action [9,30–32].

**Figure 1.**
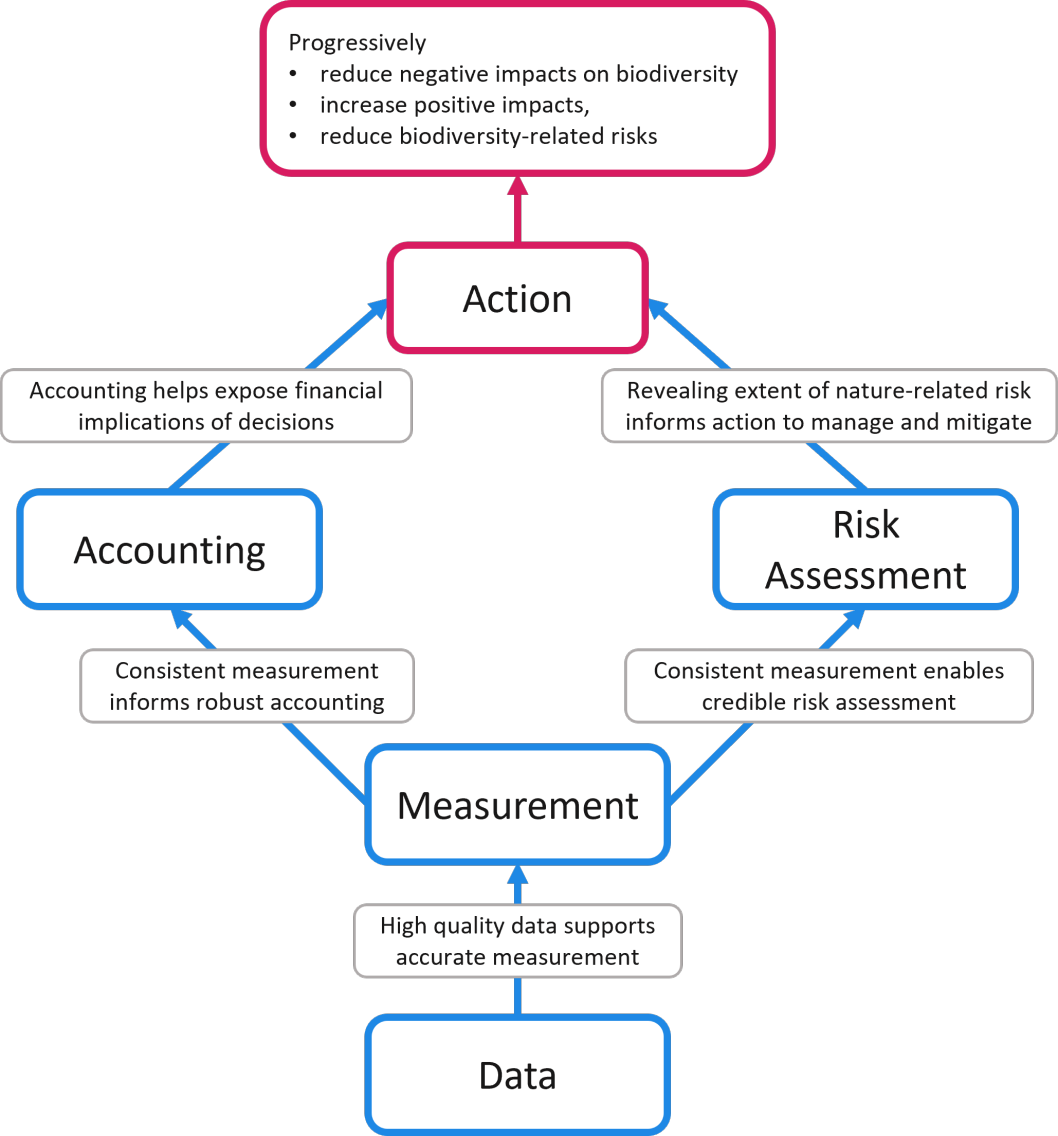
Four science and technical priorities for private sector action to address biodiversity loss.

Based on experience supporting the private sector with practical assessment approaches, datasets, methods and frameworks, we outline four science and technical advances that are necessary to enable action by the private sector at a scale that contributes to halt and reverse biodiversity loss (figure 1). First, further advances and alignment are needed to agree on a consistent set of indicators and metrics for measuring changes in the state of nature and provision of ecosystem services. Second, increased access is needed to global, regularly updated, location-specific and consistent data to support measurement against this set. Third, standardized corporate and national accounting systems are needed that structure nature data, support risk assessment and risk management and create accountability at corporate, ecosystem and national levels. Finally, integrated risk assessment approaches are needed to help corporates, financial institutions, central banks and supervisors to assess and manage biodiversity-related risks, drawing on these accounting, data and measurement systems. Credible science and technical advances in these four areas build upon each other and are required foundations for further action at scale by both businesses and financial institutions.

The science and technical priorities we set out recognize that the sustainability of humanity’s engagement with nature is about the functioning of the biosphere as a whole, including both its living and non-living components [9]. We frame biodiversity loss in the wider context of nature degradation, recognizing the interactions between plant, animal and microorganism communities and the non-living environment, which interact as functional units in ecosystems. The interactions of living and non-living constituents of the biosphere, such as in water, carbon and nitrogen cycles, are important to how businesses depend and impact on nature. Loss in biological diversity is of particular relevance to the private sector because it disrupts ecosystem processes that are fundamental to the provision of critical ecosystem services, such as pollination [33]. Non-living resources, such as water and hydrological services like water flow regulation and water purification, are fundamental to economic activity and important components of ecosystems and habitats, and therefore also important for consideration. Importantly, biodiversity loss, the degradation of ecosystems and the decline in the provision of many ecosystem services have significant consequences for business, and the financial institutions that finance, invest in, insure and facilitate economic activity by business.^1^ Businesses and financial institutions have dependencies and impacts on nature that create risks and opportunities to their organizations, including physical, transition and systemic risks [19,34].

## 2. Measurement

The urgency of action to address biodiversity loss has created the need to understand both corporate impacts and dependencies on nature, as well as the resulting risks and opportunities for organizations to inform disclosures, target-setting, transition planning and response actions [19,35–37]. When embedded within a causal framework such as driver-pressure-state-impact-response, standardized measurement approaches can support credible management and consistent reporting by businesses and financial institutions, and support broader policy and societal objectives.

The complexity of biodiversity must be reflected in meaningful assessments and identification of required response actions. Proposals of single, universal metrics to describe biodiversity loss, such as species extinction [38] and response efforts, such as area-based metrics of protected area coverage [39], appeal in their simplicity, akin to climate metrics, such as atmospheric concentration of CO_2_ and corresponding degrees of temperature warming. However, the use of single metrics may miss factors necessary to quantify biodiversity loss or gain and mischaracterize the actions needed.

While the use of any single, universal metric has inherent limitations for biodiversity, the proliferation of thousands of unique nature-related indicators and metrics, with different definitions for similar measures, has created confusion in the private sector. Report preparers regularly call for consistency in requirements across jurisdictions to increase efficiency and reduce the cost and resources required for reporting, particularly by multinational companies with complex value chains that cover many diverse locations. Investors also benefit from consistency in indicators and metrics used in reports, to enable comparison of company performance across and within sectors [19]. Some business associations have called for mandatory assessment and disclosure of nature-related issues to create a level playing field [40]. Mandatory disclosure entails some level of standardization in the methods for measurement and the indicators and metrics specified in standards and regulations.

Consistent metrics for measurement of dependencies, impacts and performance can change norms and standards against which organizations are evaluated, including by finance, investment and insurance, thereby shifting business strategy and entire sectors and industries. Agreement on a suite or ‘dashboard’ of indicators and metrics for measuring changes in the state of nature and its biodiversity, as well as the provision of ecosystem services, is ultimately needed to support the successful implementation of the GBF. The GBF calls for a ‘whole of society approach’ to implementation which includes action by business against each target and goal, and therefore the full scope of the GBF monitoring framework. The monitoring framework was adopted at COP15 through decision 15/5 [8] with a provision for an Ad Hoc Technical Expert Group (AHTEG) to undertake further work throughout 2024 and to provide advice on the operationalization of the monitoring framework at COP16 in October 2024. Several goals and targets of the GBF have well-established headline, component and complementary indicators with relevance to business. However, target 15, which addresses business disclosures, required further review of the headline, component and complementary indicators by the AHTEG, and additional work because ‘an agreed up-to-date general methodology does not exist’. The entry within decision 15/5 for the complementary indicator(s) for target 15 makes reference to being based on the Taskforce on Nature-related Financial Disclosures and the guidance document for countries mentions several times the TNFD framework as a key tool to help business to disclose [41].

Initiatives like the Science-Based Targets Network (SBTN) and TNFD have already collaborated with scientific partners to begin to address this need for consensus indicators and metrics. There has been notable progress in building consensus on the measurement of the pressures driving biodiversity loss, such as greenhouse gas emissions and–beyond climate change–metrics like water withdrawal and consumption, land use and land cover change and nutrient pollution. These indicators are all covered by TNFD, GRI, SBTN and the European Sustainability Reporting Standards. Pressure or ‘impact driver’ indicators and metrics correspond to the five drivers of nature change identified by the Intergovernmental Science-Policy Platform on Biodiversity and Ecosystem Services (IPBES) and can be directly linked to business production processes [19,22,36,42].

There is emerging consensus on a small number of indicators for the state of nature–ecosystem condition and extent [28] and species abundance and species extinction risk [15]. These indicators and metrics for the state of nature are now emphasized by the SBTN and TNFD in guidance for the private sector. These build on the work of the Group on Earth Observations Biodiversity Observation Network (GEO BON) that has characterized Essential Biodiversity Variables for global and national monitoring to support policy [43,44] and the United Nations Statistics Division in its development of the UN System of Environmental-Economic Accounting, Ecosystem Accounting—SEEA EA [28]. There are moves towards creating further consensus on a small set of metrics to evaluate changes in the state of nature, such as the consultation by the Nature Positive Initiative (NPI), a coalition of conservation organizations, business and finance coalitions, sustainability standards and target setters, indigenous knowledge and scientific institutes [45].

However, there still remains a lack of consensus on which metrics to use for these indicators and a paucity of guidance on the measurement of ecosystem services. In response, the TNFD has provided indicators for private sector disclosure of changes in the state of nature but only provides illustrations of metrics for each indicator. Similarly, the SBTN has provided metrics for corporate impact assessment and target-setting to describe both biodiversity and other components of nature. In some cases, the guidance is prescriptive but in other cases, private sector actors may choose, within defined bounds, the appropriate metric to describe impacts or guide actions. Metrics which capture, for example, local variability in species extinction risk or ecosystem integrity [46] as well as local stakeholder values and knowledge [18] will more accurately reflect site level company actions for target-setting and action than aggregate country level metrics. This means that in each methodology, the choice of metric also needs to reflect how it is used.

As with the evolution in indicators and metrics for measuring climate impacts, risks and responses, we expect a suite of consensus metrics for changes in the state of nature, and the provision of ecosystem services to evolve in response to: (i) changes in technology and data availability; (ii) the need to enable integrated accounting and accountability; (iii) emerging common use cases including in the private sector; and (iv) the need for effective, integrated risk assessment in a rapidly changing world.

Although many organizations have been acting without waiting for full consensus, further alignment on nature, biodiversity and ecosystem service measurement would accelerate and scale action. Consensus will build confidence in credible, consistent metrics that can be relied upon to inform decisions, particularly those with significant implications such as changes to strategies, policies, business models, risk management decisions and investment criteria. Consensus will also secure greater consistency and coherence in actions which are important to: (i) shift entire business sectors, by changing norms and standards against which sectors are judged (critical mass), thereby supporting transition planning and transition finance and (ii) increase the likelihood of successful interventions in landscapes, watersheds and seascapes where trade-offs, synergies and cumulative impacts need careful consideration.

A further important and connected priority is the assessment of additionality for corporate sustainability actions to meet voluntary targets like science-based targets for nature. This is now evaluated in the certification of forest carbon credits, where additionality is considered as avoidance of imminent threats of decline or loss [47], but it remains to be incorporated into the wider consideration of nature actions on value chain impacts. Business and financial institutions will increasingly want to understand and demonstrate how their actions contribute to achieving the goals and targets of the GBF. The use of counterfactual methods that can isolate the effects of business on nature outcomes could help to provide scientific rigour to alignment assessments, sustainability scores and credibility to the use of the term ‘nature positive’.

Arriving at consensus will inevitably be difficult. At the political level, consensus was not achieved on the full suite of metrics within the GBF monitoring framework for adoption at COP15. The need to find consensus is implicit in the invitation extended within decision 15/5 to several different groups, bodies and organizations to support the operationalization of the monitoring framework [8]. One such body is the IPBES whose government members decided in 2023 to initiate a methodological assessment on ‘monitoring biodiversity and nature’s contributions to people’. The scope for this assessment was approved by the 10th IPBES Plenary in 2023 and includes analysis of the data and systems that are ‘available and needed’ [48]. This assessment will complement the methodological assessment of ‘the impact and dependence of business on biodiversity and nature’s contributions to people’ [49], which includes a chapter on data needs and approaches for the measurement of business performance. This prioritization of IPBES assessments on these topics reinforces that consensus on these topics has not yet been reached.

## 3. Data

To enable this measurement, access is needed to global, regularly updated, location-specific and consistent data on the drivers of nature change, changes in the state of nature and biodiversity, and the provision of ecosystem services. Broad access to data with global coverage and high temporal and spatial resolution is a necessary enabler for measurement and action by the private sector, as well as governments, international organizations and communities. As with the identification of consensus indicators and metrics, the data needed to inform effective and equitable interventions for biodiversity loss are multi-faceted, because it is complex, needs to be understood at a system level, and the causes of biodiversity loss are diverse.

The availability of data on drivers of nature loss and changes in the state of nature has improved dramatically over the past decade, with advances in remote sensing (such as the European Space Agency Sentinel mission and datasets) and novel data infrastructure (such as the Global Biodiversity Information Facility). This is already enabling more accurate monitoring and evaluation, particularly at the ecosystem level. Increased use of dynamic data is improving the responsiveness of solutions to accelerating drivers of biodiversity loss, such as resource extraction and climate change-induced species range shifts [50]. However, despite this growth in data availability, clear and persistent gaps exist in the coverage necessary to mitigate biodiversity loss [51–53]. These include gaps in taxonomic and geographic coverage, particularly for threatened species and ecosystems disproportionately impacted by anthropogenic pressures, as well as on key threats to biodiversity and ecosystem services. Gaps in data availability in the global tropics exacerbate threats to areas of high biodiversity richness and vulnerability, and also have consequences for Indigenous Peoples and local communities who may be most affected by company activities and biodiversity loss.

Where data exist, they are not always accessible or fit for purpose for use in the private sector. Traditional biodiversity data repositories often contain either primary site level data or aggregate, static data. Dynamic, secondary data sources fit for corporate use are needed to reduce barriers to sustainability reporting, target-setting and action [54]. The need to increase the availability of data for decision-makers is recognized through target 21 of the GBF [7].

The collection and management of biodiversity data has been historically underfunded. Best estimates suggest that $114 million is required to reach pre-defined baselines for coverage across four global biodiversity spatial datasets [55]. Yet systematic underfunding of biodiversity data is evident, for example in the call of the IUCN for funding to support their Red List of Threatened Species [56]. This dataset in turn underpins other biodiversity datasets such as the World Database on Key Biodiversity Areas and the Critical Habitat screening layer. Conservation spatial data have not typically been collected and collated with the intention to inform corporate or financial decision-making. As a result, the infrastructure needed to underpin fit-for-purpose biodiversity data in this context has also been underfunded. The need for open access and high-quality data but without sustainable sources of funding for data collection, storage, analysis and dissemination leads to two contrasting problems. First, lack of funding leads to incentives for actors across the data value chain to apply restrictions for commercial purposes to retain the ability to generate funds from licensing of data. Second, it can result in data being made freely available, with no provision for updates or other essential maintenance to ensure that its continued provision is of a high quality. Further, the private sector is rapidly emerging as a producer of nature-related data, not just a consumer, for example of eDNA data. This increases the challenge of harmonization, connection and open access and the need for standards, protocols and incentives for data quality and accessibility.

Historic funding challenges are further exacerbated by the increasing need for bottom-up data as a result of the focus within the GBF and its monitoring framework on national and regional action. Central to the monitoring framework is the role of monitoring and review based on national level information, which will require a significant shift in the systems and capacity (and therefore funding needs) at the national and regional levels. It is clear that the need at the national level in combination with the existing priorities for the private sector outlined above cannot be met without changing the approach to funding for biodiversity data. This conclusion was the basis for a dialogue convened by the UN Environment Programme World Conservation Monitoring Centre (UNEP-WCMC) with the European Commission and the Moore Foundation to explore a new approach to sustainable financing of biodiversity data [57]. The dialogue recognized that funding for data has typically been project based (and therefore short term), dependent on donors (and therefore subject to changes in priorities of funding organizations), reliant on core funding from government (and therefore exposed to shifting political priorities) or contingent on funding from NGOs (and therefore coupled with the financial sustainability of individual organizations). Even the existing funding to biodiversity data may be at risk as a result of efforts by new and existing commercial entities to create products and services on biodiversity, without ensuring that the necessary data underpinning their products and services are well funded. For example, since August 2022, 116 requests have been made to the Integrated Biodiversity Assessment Tool Alliance for access to one or more spatial biodiversity datasets. Of these, 87% have been for start-ups, consultancies, environmental social governance product providers or other commercial uses that would not automatically support the further development of biodiversity data (personal communication from Matt Jones, 2024). The increase in interest and willingness to pay by the private sector for products and services built on biodiversity data may not result in increased funding for that data without intervention.

Three foundational steps could help to address these issues. First, work is needed to scope how relevant data should be collected, maintained and made available, and how the funding issues could be addressed. Second, given that significant data already exist [58], an immediate priority is to ensure datasets are discoverable and accessible to new audiences, by mapping and cataloguing existing data. A third priority is the identification of data gaps that need to be filled to support assessment, target-setting, reporting and transition planning by businesses and financial institutions, building from emerging demand for this data, real use cases and existing gap analyses [53,59].

One proposition is for a global nature-related public data facility that would provide a focal point for data access [54,59]. This could involve government, scientific, private sector and civil society actors contributing to a collective good solution at a global scale, contributing different expertise and data availability into a common use platform. This could be supplemented by national and sub-national initiatives that can be aggregated and linked into the global facility. Several governments have, or are developing, data platforms. These can help address user needs, but national level solutions alone may be insufficient, with ongoing challenges in standardization and comparability of datasets, and high transaction costs for users operating across jurisdictions. A global nature-related public data facility–with carefully determined scope, governance, financing and incentive structures, and supported by data and measurement standards—could expand the availability of biodiversity data and insights with significant benefits for public, private and civil society.

A feasible and sustainable funding model for nature-related data requires further consideration, including in relation to long-term commitments of public funding coupled with private investment, for example with a proportion of profits reinvested from commercial analytics based on core biodiversity data to support its provision.

To improve discoverability and accessibility of global and national data, clear frameworks are needed for what data need to be collected and how these should be maintained. Data standards are needed to improve consistency in datasets and data platforms [59]. Finally, a process will be needed to build a shared understanding of, and agreement on, common priorities among businesses and financial institutions, the biodiversity data and analytics community and other stakeholders. The alignment of priorities for data collection in these communities will need to balance the requirements of corporate users, scientists and researchers and broader society for biodiversity assessment and monitoring. This can benefit from learning from case study examples to identify user needs, priorities, assumptions and possible trade-offs [59]. Data will need to support measurement using consensus indicators and metrics for particular use cases. And these indicators and metrics will need to be tested to understand how robust they are in the face of existing and likely enduring data gaps.

Identifying and filling gaps in data coverage would support a more comprehensive assessment of corporate impacts and dependencies on nature. At present, data for species have significant taxonomic gaps, notably for plants, although progress is being made with the threat status of over 70% of tree species now assessed (IUCN Red List of Threatened Species, v. 2023-1). Given the number of data gaps, prioritization will be needed for those that address common user needs and align with consensus metrics and indicators for measurement. Additionally, advances must continue to address the challenge of expanding taxonomic coverage while improving geographic coverage and spatio-temporal resolution. This includes scaling existing efforts (for example the identification of Key Biodiversity Areas or the development of spatial data to support the IUCN Red List of Ecosystems) as well as funding novel research, innovation and data development.

Given the expense of collecting primary biodiversity data through traditional field surveys and the growth in demand for biodiversity data, new technologies are emerging that can increase the efficiency of data collection, generation and analytics. Novel approaches include the application of technological monitoring solutions (such as satellite-based remote sensing, cameras, acoustic recording devices and environmental DNA testing) [60,61] as well as citizen science data collection (e.g. iNaturalist or eBird) that may be more feasible to scale and support by alternative funding mechanisms (e.g. crowdfunding or corporate or philanthropic funding). These innovations present an exciting opportunity to improve data generation, but work is needed to ensure these data are consistent, comparable, credible and can be connected across datasets to support interpretation and use by the private sector. To interpret and appropriately use these datasets, for example from acoustic monitoring and large citizen science initiatives, and the outputs of remote sensing and other innovative technologies, still requires significant technical expertise and post-processing. Finally, collection, processing and dissemination of priority data for ecosystem services are required to help business and finance users draw the connection between biodiversity and nature-related dependencies, impacts and risks.

A further important challenge at the interface of measurement and data is corporate value chain traceability. Many businesses and financial institutions do not know where, in terms of specific locations, they are sourcing from or where their downstream impacts occur. Accurate geospatial information on where corporate assets and activities are located along value chains is needed to complement the improvements in biodiversity measurement outlined above. This is required to support high quality, accurate impact and risk assessments and related disclosures, where material impacts and risks may occur far upstream or downstream. Advances in geospatial asset location data, value chain traceability and biodiversity data are all needed.

## 4. Natural capital accounting

Standardized and consistent accounting systems that structure data, support risk management and create accountability at corporate, ecosystem and national levels, are needed to enable the integration of biodiversity into relevant decision-making. This is recognized in target 14 of the GBF which acknowledges the importance of integrating the values of biodiversity and opportunities for conservation and sustainable use of biodiversity into decision-making across all sectors, including the private sector, to align their relevant actions and financial flows with the GBF [7]. One important approach to support this, especially at the national level, is natural capital accounting that integrates information on nature, the economy and their linkages to provide consistent, comprehensive, standardized and regularly produced information on the state of nature and its contributions to the economy and human well-being [28,62].

Natural capital accounting views nature as a form of capital: a stock of assets that support a flow of benefits to people now and in the future [63]. By integrating nature and economic statistics, natural capital accounting helps fill the absence of nature and biodiversity in key economic indicators for public and private sectors alike. Earnings and share prices are key indicators of corporate performance, and GDP continues to serve as the headline indicator of countries' performance including macroeconomic status and trends. Natural capital accounting aims to correct the fundamental failure of these measures, and consequently many economic decisions, to integrate nature, including the contributions of nature to economic activities and the impacts of economic activities on nature, and ultimately to contribute to measures of inclusive wealth [9,64].

**Figure 2.**
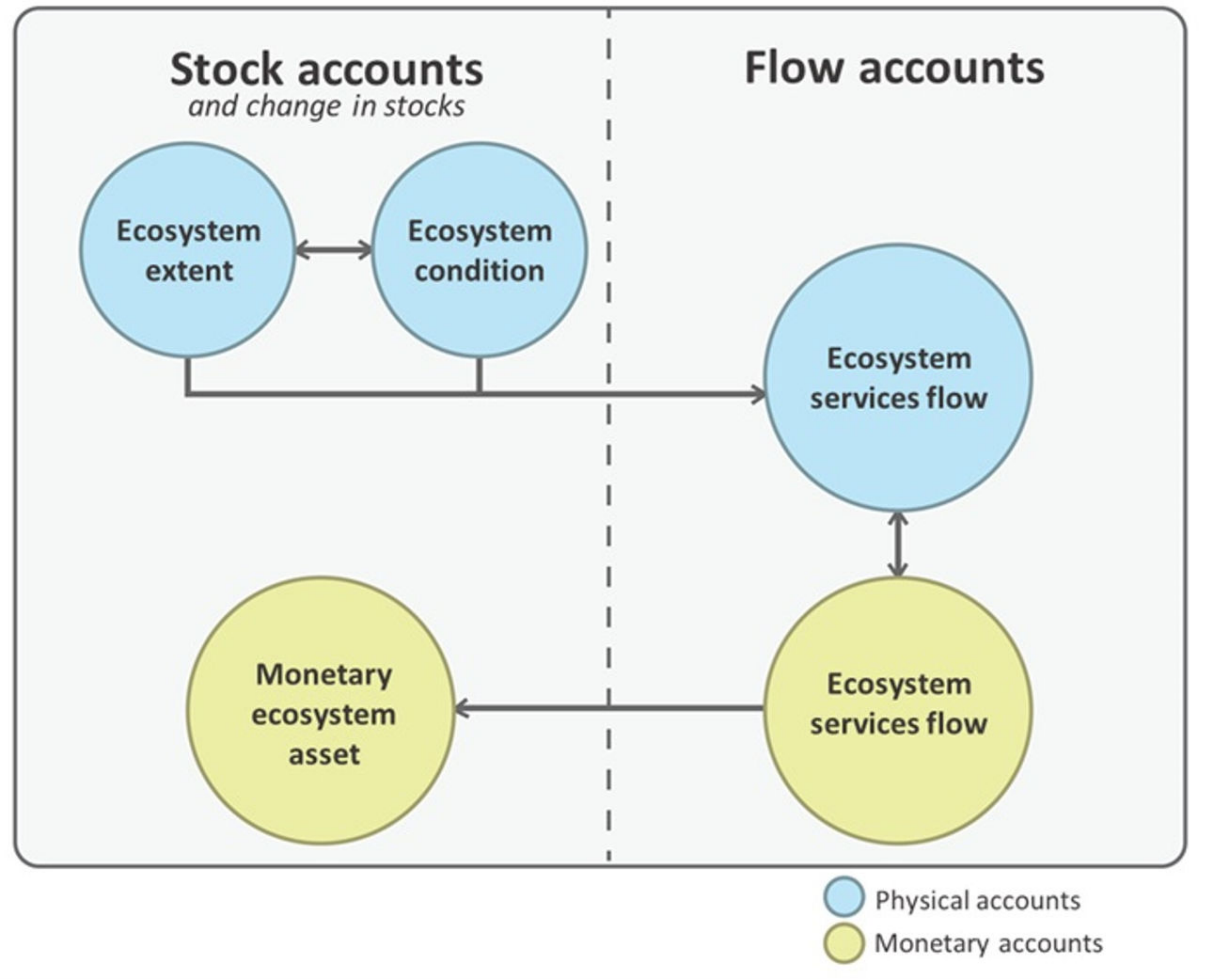
Ecosystem accounts and their connections under the SEEA EA. Source: United Nations [28]. From *System of environmental economic accounting—Ecosystem accounting (SEEA EA)*, by SEEA EA. ©United Nations 2021. Reprinted with the permission of the United Nations.

Natural capital accounting has progressed rapidly over recent years. In the corporate world, it has emerged as a key process and tool to support businesses to identify, assess and manage their actions related to nature [16,65,66]. In the national accounting context, a key advance is the development of the UN System of Environmental-Economic Accounting, Ecosystem Accounting (SEEA EA). It was prepared under the auspices of the United Nations Statistical Commission, using inputs from hundreds of experts in fields such as accounting, economics, environmental science and statistics, and adopted as an internationally agreed statistical framework in 2021 [62]. The SEEA EA describes ecosystems and benefits they provide to the economy and to people, using approaches consistent with the System of National Accounts (SNA). The SEEA EA specifies a set of five ecosystem accounts (figure 2), including three that are compiled using physical metrics (ecosystem extent, ecosystem condition and ecosystem services physical accounts) and two that are monetary accounts (ecosystem services monetary accounts and ecosystem asset accounts).

While the adoption of the SEEA EA as an international statistical standard presents an important milestone in natural capital accounting, it merely paves the way for the necessary further steps, starting from scaling-up implementation [62,67]. This requires engagement with different stakeholders, such as the business community, along with communication, advocacy, data and tools development, capacity building and coordination.

Monitoring progress towards the achievement of biodiversity-related goals and targets is another area where a comprehensive and robust measurement framework for natural capital accounting can be informative. This is recognized by the monitoring framework for the GBF [8] that notes the value of aligning national monitoring with the SEEA to help mainstream biodiversity and strengthen monitoring systems and reporting. The same decision also notes the importance of aligning GBF indicators with existing inter-governmental processes such as SEEA EA. Accordingly, in developing guidance for the operationalization of the GBF monitoring framework, AHTEG directly draws from SEEA EA to develop indicators A.2 (extent of natural ecosystems) and B.1 (services provided by ecosystems) [68].

The business community itself has pioneered and is actively engaged in natural capital assessment and accounting, including some of the early development and testing of the Natural Capital Protocol and related metrics [16,69]. Important steps for development and standardization have been reached on a national level with the adoption of the SEEA EA, but further alignment of corporate accounting systems with SEEA EA is needed to ensure the coherence between private, public and financial accounting systems [66].

Consistency between accounting methods and metrics applied by different organizations, including in public and private sectors, is not a need unique to natural capital accounting but rather, a principle more generally applicable, advantageous and necessary to consistently measuring and monitoring activities and their outcomes across different actors and activities at different geographic and temporal scales. In economic accounting, the compilation of the SNA draws extensively from financial and other information that originates directly from business and financial sectors and their recordkeeping [70,71]. This enables comparability of reported economic activities and outcomes by different actors and also reduces the combined reporting burden required for the compilation of different accounts. As such, business accounts, alongside business surveys, represent a major source of data on national accounts on corporate activities. While achieving perfect correspondence of national and business accounts may require specific adjustments to data, consistency of business accounts with national accounts is a necessity [70,72]. Similar principles apply also to accounting for greenhouse gas emissions; for example, the GHG Protocol Corporate Standard [73] and the ISO standard 14 064 for GHG emission inventories [74] emphasize the consistency of their tools and approaches with guidance by the IPCC for the compilation of emissions data at national and global levels.

Although a national standard has been introduced in the United Kingdom [75] and a global standard is under development [76], there is currently no formal global standard for corporate natural capital accounting, especially when considering alignment with SEEA EA. While the SEEA EA focuses on national and sub-national accounting, many of its basic principles are applicable, with proper adaptation, to natural capital accounting for corporate actors [66]. On the other hand, it is not obvious at the outset that SEEA EA and its five accounts will directly translate into an operational accounting framework applicable for corporations managing complex operations, including domestic and international value chains. As such, the integration of SEEA EA concepts and principles into corporate level accounting is a key area of further research and development in natural capital accounting.

The process of compiling natural capital accounts itself helps develop further knowledge about the links between nature and the economy, and the accounting framework and its principles must be evaluated and evolve continuously. The SEEA EA framework already identifies several issues for further research, including the treatment of climate regulating services, issues related to the valuation of ecosystem services and ecosystem assets, and ensuring consistency with the SNA [28]. As noted above as a priority for measurement, another important area of further work involves accounting for biodiversity at its different levels, including ecosystem, species and genetic levels [77]. More generally, incorporating ecological principles, including complex system properties of the natural environment, into natural capital accounting remains a high priority [63], as well as accounting at ecosystem levels to support effective management [78]. Moreover, the development of methods for the identification, assessment and management of nature-related financial risks remains a key area of research [79,80], including how information from the SNA and natural capital accounts can best support operationalization of such approaches.

## 5. Risk assessment and management

There are two further connected and important routes to achieving target 19 of the Global Biodiversity Framework, to substantially and progressively increase the level of financial resources for biodiversity [7]. The first involves concentrating on the quantum of finance being directed towards activities to conserve and restore nature. The second calls for a more realistic pricing of nature-related financial risks in the belief that such pricing will drive increased capital requirements for activities that harm nature and, as such, by default will direct financing away from those activities and towards less capital-intensive activities that enhance nature [34]. Both require assessment and quantification of such risks. And while there are existing frameworks to understand and categorize nature-related dependencies and impacts on a sectoral and activity basis [37], the assessment and quantification of nature-related financial risks for a particular business, be it corporate or financial, is not yet widely available. The development of corporate assessments of nature-related risks is of particular importance, as financial institutions ultimately rely on corporate assessments to understand risks inherent in their portfolios.

Within mainstream risk management, the typical flow of risk management action incorporates risk identification, risk assessment, risk mitigation and, ultimately, client engagement. The first three pillars are equally relevant for corporates and financial institutions. Within the risk identification pillar, there are a number of methodological frameworks that are targeting strategic reviews, heat-mapping approaches as well as total exposure estimations [37, annex 2). These frequently spring from an analysis of dependencies on ecosystem services and include, but are not limited to, the use of tools such as ENCORE [81] and the WWF Risk Filter suite [82]. These have already been used on financial portfolios at the level of financial institutions [83] as well as at the national level [84–86]. The Network for Greening the Financial System has developed a draft conceptual framework for how nature-related financial risks can be addressed by central banks and financial supervisors [79], and the Organisation for Economic Co-operation and Development (OECD) has developed a prudential framework for assessing such risks [87].

Currently, the risk assessment pillar requires the most attention. Within mainstream risk management, risk assessment encompasses approaches such as scenario analysis, stress testing, probabilistic modelling and incorporation into credit and market risk models. Scenario analysis and stress testing dominate the first published risk assessments. Most of these fit into micro-prudential approaches, where the impact of a particular scenario (be it an introduction of a policy or an extreme event) is analysed on a firm or a supply chain [88,89]. There are also emerging methodologies that are considering incorporating nature-related indicators within macroprudential scenarios, which enable economy-wide stress testing [80]. One of the key elements of the further development of scenario methodologies is the need for integrated climate–nature nexus scenarios to capitalize on a more systematic assessment of the nature–climate nexus [80,90,91].

To the extent that nature-related extreme events are already being assessed within the natural catastrophe models of the insurance market, these can be used as examples of probabilistic modelling. However, to be able to assess nature-related financial risks in their day-to-day decision-making processes, financial institutions urgently need more examples of how such scenario approaches might work as well as new non-scenario based risk assessment methodologies across the insurance, banking and investment industries.

Ultimately, more sophisticated and standardized risk assessment approaches need to be developed to enable financial risk mitigation to be conducted at scale across the financial industry. Usually, risk mitigation entails introducing limits and monitoring compliance with such limits. Equally, as noted above, there are indicators and metrics of biodiversity loss, which can already be tracked today. And while global cross-industry agreement would be helpful in this space, action is possible now.

Finally, client engagement is perhaps the most important pillar of the risk management flow. Crucially, there are approaches to active ownership within the investment management industry, where examples are already available. Similarly, there are existing approaches within banking and insurance where nature can be built into the climate engagement process [92]. Action can take place simultaneously with further development of risk assessment, measurement and management methodologies, and collaboration across the financial, corporate, academic and NGO communities is key to driving identification, assessment and management of both risks and opportunities.

## 6. Conclusion

As recognized in the GBF, halting and reversing biodiversity loss will require the whole of society to take action, with a critical role for the private sector. Four science and technical priorities discussed here need to be addressed to scale private sector assessments, target-setting, transition planning, reporting and action: a consensus set of indicators and metrics for measuring changes in the state of nature and ecosystem service provision; improving availability and access to global, regularly updated, location-specific and consistent nature data; further development of accounting systems that structure data and support risk management and accountability; and helping businesses and financial institutions to translate science to action through integrated risk assessment.

## Data Availability

This article has no additional data.

## Footnotes

^1^
Also governments through sovereign financing, but this is beyond the scope of this article.
